# Global Regulatory T-Cell Research from 2000 to 2015: A Bibliometric Analysis

**DOI:** 10.1371/journal.pone.0162099

**Published:** 2016-09-09

**Authors:** Yin Zongyi, Chen Dongying, Li Baifeng

**Affiliations:** 1 Department of Hepatobiliary Surgery and Organ Transplantation, The First Hospital of China Medical University, Shenyang, China; 2 Department of Anesthesiology, The First Hospital of China Medical University, Shenyang, China; McGill University Health Centre, CANADA

## Abstract

We aimed to analyze the global scientific output of regulatory T-cell (Treg) research and built a model to qualitatively and quantitatively evaluate publications from 2000 to 2015. Data were obtained from the Web of Science Core Collection (WoSCC) of Thomson Reuters on January 1, 2016. The bibliometric method and Citespace III were used to analyze authors, journals, publication outputs, institutions, countries, research areas, research hotspots, and trends. In total, we identified 35,741 publications on Treg research from 2000 to 2015, and observed that the annual publication rate increased with time. *The Journal of Immunology* published the highest number of articles, the leading country was the USA, and the leading institute was Harvard University. Sakaguchi, Hori, Fontenot, and Wang were the top authors in Treg research. Immunology accounted for the highest number of publications, followed by oncology, experimental medicine, cell biology, and hematology. Keyword analysis indicated that autoimmunity, inflammation, cytokine, gene expression, foxp3, and immunotherapy were the research hotspots, whereas autoimmune inflammation, gene therapy, granzyme B, RORγt, and th17 were the frontiers of Treg research. This bibliometric analysis revealed that Treg-related studies are still research hotspots, and that Treg-related clinical therapies are the research frontiers; however, further study and collaborations are needed worldwide. Overall, our findings provide valuable information for the editors of immunology journals to identify new perspectives and shape future research directions.

## Introduction

Sakaguchi [1] first described regulatory T cells (Tregs) in 1995 and reported that a few CD4+ and CD8+ T lymphocytes in normal non-immunized adult mice express IL-2 receptor α-chain (CD25) molecules, and also that the depletion of CD25+ cells leads to graft-versus-host-like wasting disease. Expression of the forkhead box transcription factor (Foxp3) is considered as the most sensitive Treg marker [2] associated with the Treg suppressive phenotype. Although the first studies focused on the inhibition of effector T-cell (Teff) priming by Tregs in secondary lymphoid organs, it has been reported that the Treg response adapts to the immune response (i.e., Th1, Th17, Th2, and Tfh cells), and that Tregs show further specialization in peripheral tissues via tissue repair and homeostasis [3–6]. In animal models, Tregs play a central role in promoting and maintaining allograft tolerance [7–9]. Kim and Lahl et al. [10, 11] demonstrated that Tregs participate in the maintenance of immune self-tolerance and homeostasis. A mechanistic function for Tregs in allergen immunotherapy was also reported [12]. A Treg vaccination was designed, aiming to treat autoimmune Type-1 diabetes [13], whereas the manipulation of Tregs as a new therapeutic strategy for various conditions, including transplant rejection, autoimmunity, and cancer, has gradually evolved [14–20].

Numerous journals have published articles on Tregs; however, only limited attempts have been made to systematically analyze the data of publications on Treg research. Bibliometric analysis has been widely used in various areas to estimate the productivity of institutions, countries, and authors; identify international collaborations and geographic distributions; and explore research hotspots and frontiers in specific fields [21, 22].

Here, we employed bibliometric analysis to qualitatively and quantitatively evaluate global Treg studies from 2000 to 2015. Our objectives were to estimate the global scientific outputs of Treg research and identify trends and hotspots.

## Methods

Data were obtained from the Web of Science Core Collection (WoSCC) of Thomson Reuters [23] on January 1, 2016. The WoSCC, including both the Social Sciences Citation Index and Science Citation Index-Expanded databases, is the most frequently used source of scientific information [24]. The terms ‘Treg*’ and ‘regulatory T cell*’ were used to retrieve titles, keywords, author information, abstracts, and references from 2000 to 2015.

The impact factor (IF) of each journal was obtained from the Journal Citation Reports Science Edition 2015, accessed on January 1, 2016 [25]. CiteSpace III (64 bits) [23, 26] was used to analyze publication outputs and construct knowledge maps. Citespace III (64 bits) is a visualization tool that can analyze scientific literature retrieved from WoSCC [23] and enables the knowledge areas to be explored through visualization and network modeling [27]. One of the most important functions of Citespace III is the detection of betweenness centrality of nodes (authors/countries/institutions/references) [28]. Betweenness centrality is an indicator of a node’s centrality in a network and is equal to the number of shortest paths from all vertices to all others that pass through that node. A node with high betweenness centrality has a large influence on the transfer of items through the network [27, 29, 30]. Collaborations (links between the nodes in graphs) were evaluated when at least one author was from a different institution and country [25]. Link thickness between two points (authors/countries/institutions/references) increases with the level of association of scholarly research, whereas the node size is related to author, institution, and reference importance [23]. A node with a purple circle shows that the centrality is > 0.1 [31]. Keywords generated by CiteSpace III capture the core concept of clusters and provide a multi-faceted overview of a knowledge domain and its associated network [27]. Additionally, CiteSpace can identify individual networks of occurrence or citation in articles published in a given time interval, known as a time slice, and then merge them to form a general picture that visually shows how a scientific field has been evolving over time. In this paper, an individual network is derived from the 50 most cited articles published in a one-year time slice [29, 32, 33]. Additionally, TF*IDF weighting was used to analyze the content of each cluster. TF*IDF is a statistical algorithm, and higher TF*IDF values indicate a stronger the ability to predict theme [34]. Finally, we applied burst detection to investigate the growth rate of citations or keywords [27]. When the number of articles with a term in their titles or abstracts sharply increased at a much faster rate than other terms, the term was defined as a burst word [27, 29, 33].

## Results and Discussion

### Analysis of publication outputs

From 2000 to 2015, 11 document types were found in 35,741 publications, including research articles, review articles, meeting abstracts, and proceedings. Most publications were research articles (61%), followed by review articles (19%), and meeting abstracts (15%). Approximately 99.7% of the publications were written in English, indicating that English is the primary language used among scholars, whereas the remaining 0.3% of the publications were written in other languages, including French, German, and Polish (data not shown).

**Fig 1:**
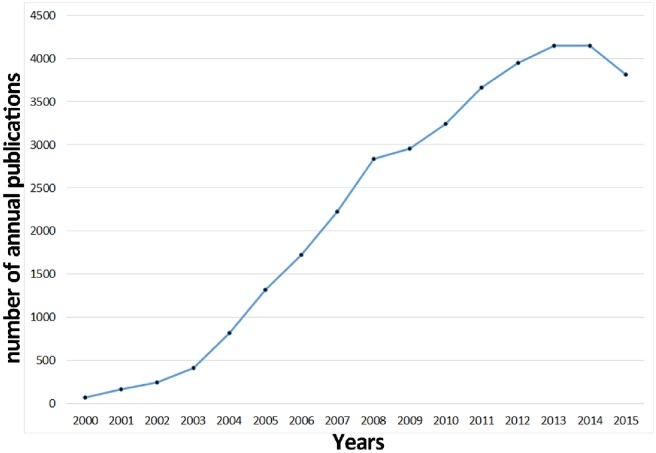
Trends in the number of scholarly publications related to regulatory T cell (Treg) research from 2000 to 2015.

The total number of publications continually increased over time, but some fluctuations were observed with regard to the growth rate of publications. Thus, the distribution of publications was divided into different time stages (Fig 1). Treg research was initiated in 2000–2003, whereas an accelerated increase occurred in 2003–2008; the number of publications was 7-fold higher in 2008 (2,833 articles) compared with that in 2003 (413 articles). Compared with the past five years (2003–2008), the growth rate of publications suddenly decreased in 2009, which might be attributed to the discovery of Th17 [35–37], which is a novel CD4+ cell subset along with Th1, Th2, and Treg. This discovery distracted the attention of Treg investigators in the following three years. However, the growth rate of publications in the years after 2009 partially recovered, which could be attributed to the discovery [38, 39] that Th17 and Treg have reverse bio-functions, and that a change in the Th17/Treg ratio can improve autoimmune and inflammation diseases. Thus, numerous clinical and basic studies focused on this mechanism. In addition, the continual increase in the total number of publications might have resulted from the increased number of journals indexed in the WoSCC database. In 2015, the growth rate of publications initially declined, probably because of the follows: 1) some investigators finished their projects on Treg cells and changed their research orientation; and 2) some papers published in late 2015 could not be retrieved because of an approximately four-week delay in WoSCC updating [40].

### Journal analysis

More than 324 scholarly journals have published articles on Treg research. Bradford’s Law of Scattering is a pattern first described by Samuel C. Bradford in 1934 that estimates the exponentially diminishing returns of extending a search for references in science journals. One formulation is that if journals in a field are sorted by the number of articles into three zones, each with approximately one-third of all articles, then the number of journals in each zone will be proportional to 1:n:n^2^. [41, 42]. Using this classification, we found that 2% (seven journals) of the journals that published articles on Treg research were distributed in zone 1, 14% (45 journals) of the journals were distributed in zone 2, and 84% of the journals (272 journals) were distributed in zone 3, which had a lower influence than zone 1 or 2 (Table 1).

**Table 1:** Bradford’s Law of Scattering for journals that published articles on regulating T cell (Treg) research from 2000 to 2015.

	n	n/N(%)
Zone 1	7	2.2
Zone 2	45	13.9
Zone 3	272	84.1

Notes: n, the number of journals in zone 1, 2 or 3; N, the number of all journals.

The 15 most active journals are presented in Table 2. *The Journal of Immunology* (IF2015, 4.922) published the highest number of articles on Treg research (2,346 articles, 7%), followed by *PLoS One* (IF2015, 3.234; 1,078 articles, 3%), *Blood* (IF2015, 10,452; 907 articles, 3%), and the *American Journal of Transplantation* (IF2015, 5.683; 721 articles, 2%). Additionally, the *Journal of Clinical Immunology* (IF2015, 3.184) devoted 492 (25%) of its publications to Treg research, followed by *Immunology* (IF2015, 3.795; 584 articles, 21%); *Cancer Immunology*, *Immunotherapy* (IF2015, 3.941; 280 articles, 16%); and the *American Journal of Transplantation* (IF2015, 5.683; 721 articles, 16%). Compared with other journals, articles on Treg research were more likely to be accepted by these active journals.

**Table 2:** The top active 15 journals that published articles on regulatory T cell (Treg) research from 2000 to 2015.

Ranking.	Journal	Country	N1 (%)	N2	N1/N2	IF2015
1	J IMMUNOL	USA	2,346 (6.56)	18,330	12.8	4.922
2	PLOS ONE	USA	1,078 (3.03)	450,600	0.2	3.234
3	BLOOD	USA	907 (2.55)	12,795	7.1	10.452
4	AM J TRANSPLANT	Demark	721 (2.03)	4,485	16.1	5.683
5	EUR J IMMUNOL	USA	648 (1.82)	4,830	13.4	4.034
6	IMMUNOLOGY	UK	584 (1.64)	2,730	21.4	3.795
7	J CLIN IMMUNOL	USA	492 (1.38)	1,935	25.4	3.184
8	TRANSPLANTATION	USA	391 (1.10)	5,640	6.9	3.828
9	J ALLERGY CLIN IMMUNOL	USA	366 (1.03)	4,740	7.7	11.476
10	J INVEST DERMATOL	USA	356 (1.00)	4,155	8.6	7.216
11	P NATL ACAD SCI USA	USA	347 (0.97)	53,685	0.6	9.674
12	CLIN EXP IMMUNOL	UK	334 (0.94)	4,260	7.8	3.037
13	CANCER IMMUNOL IMMUN	USA	280 (0.79)	1,725	16.2	3.941
14	IMMUNITY	USA	277 (0.78)	2,235	12.4	21.561
15	J EXP MED	USA	277 (0.78)	2,820	9.8	12.515

Notes: N1 (%), total number of Treg-related articles in a journal from 2000 to 2015; N2, cumulative number of articles in a journal from 2000 to 2015; N1/N2, the ratio of the total number of Treg-related articles to the cumulative number of articles from 2000 to 2015; IF 2015, impact factor of the journal in 2015.

Finally, the impact factor (IF) of a journal is an important factor in evaluating its value and that of included articles. Herein, we explored the association between the IF (2015) of journals and the number of articles on Tregs. Of the top active 15 journals, more than 25% (4/15) of the journals, including *Immunity* (IF2015, 21.561), the *Journal of Experimental Medicine* (IF2015, 12.515), the *Journal of Allergy and Clinical Immunology* (IF2015, 11.476), and *Blood* (IF2015, 10.452) had an IF > 10.000; approximately 50% (7/15) had an IF > 5.000, and all of them had an IF > 3.000 (Table 2). At least 7% of the total number of articles in the journals with an IF > 10.000 were Treg-related articles, which accounted for 5% of the total number of Treg-related articles. Additionally, the journals with 5.000 < IF < 10.000 published 4% of the total number of Treg-related articles, and the journals with 3.000 < IF < 5.000 published 17% of the total number of Treg-related articles. In summary, comparing the rate of Treg-related articles high-IF journals to that of all journals (rate of journals with IF > 10.000, 2%; IF > 5.000, 7%; IF > 3.000, 20%; and IF < 3.000, 80%) [43], Treg-related articles were relatively intensively published in high-IF journals.

### Country and institution analysis

Distribution maps provide valuable information and help researchers to identify potential collaborators. The graphed links between institutions/countries represent collaborations. As shown in Table 3, countries and institutions engaged in Treg research were distributed worldwide.

**Table 3:** Ranking of countries and institutions that published articles related to regulatory T cell (Treg) research from 2000 to 2015.

Ranking	Frequency	Country	Frequency	Institution
1	11,745	USA	917	Harvard Univ
2	3,110	China	415	Univ Pittsburgh
3	2,987	Germany	341	Univ Oxford
4	2,138	UK	331	INSERM
5	2,054	Japan	317	Univ Penn
6	1,615	France	300	Univ Calif San Francisco
7	1,612	Italy	284	NIAID
8	1,143	Netherlands	278	Stanford Univ
9	986	Canada	256	Univ Washington (Seattle)
10	920	Australia	251	Kings Coll London

#### Country analysis

**Fig 2:**
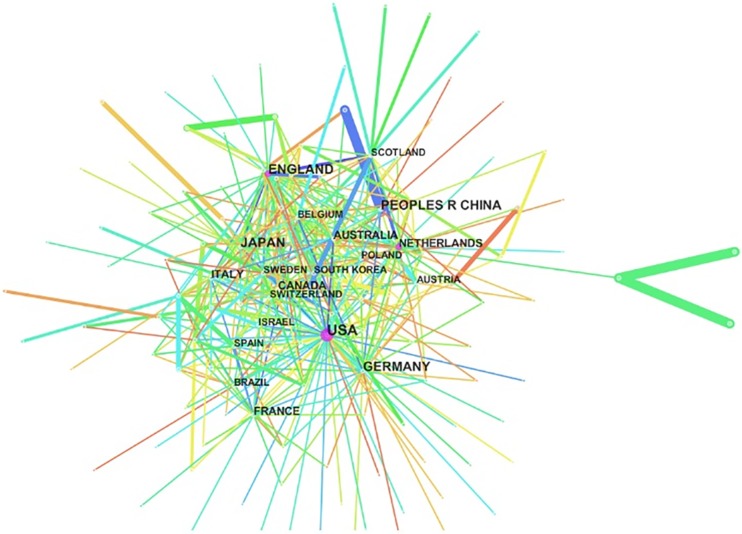
Map of countries and territories of groups that published articles related to regulatory T cell (Treg) research from 2000 to 2015.

The 35,741 articles on Treg research were published by research groups in 109 countries/territories (Fig 2). The top 10 countries (five European countries, two Asian countries, two American countries, and Australia) published 34,725 articles, accounting for 79% of the total number of publications. Along with China, which was the only developing country in this group, indicating its evident progress in life sciences in the past few years, the USA, Germany, UK, and Japan were at the top of the list. USA (11,745 articles) and China (3,110 articles) were the top two countries, accounting for 42% of the total number of publications. Furthermore, strong collaborations were identified between the UK and Canada, USA and Australia, and France and Italy.

#### Institution analysis

**Fig 3:**
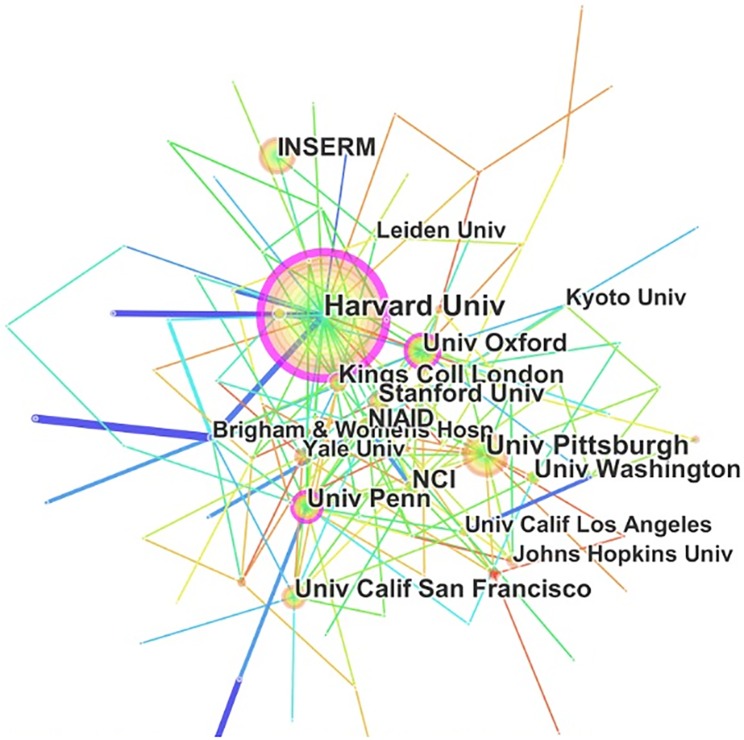
Institutions that published articles related to regulatory T cell (Treg) research from 2000 to 2015.

The 35,741 articles on Treg research were published by more than 200 research institutions (Fig 3). The top 10 institutions published 3,690 articles, accounting for 10% of the total number of publications. The first principal research echelon was led by Harvard University, followed by University of Pittsburgh, Oxford University, and the Institut National de la Santé et de la Recherché Médicale (INSERM). Furthermore, the strongest collaborations were identified between Harvard University and Brigham and Women’s Hospital, and between the University of Washington-Seattle and the University of California-Los Angeles.

### Author analysis

**Fig 4:**
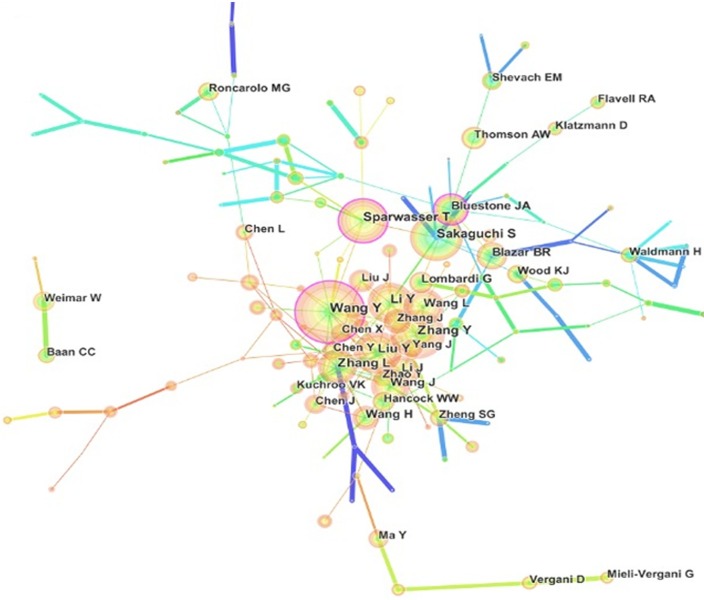
Map of authors who published articles related to regulatory T cell (Treg) research.

The 35,741 articles on Treg research were drafted by more than 30,000 authors (Fig 4). The top 15 authors are presented in Table 4 along with the number of publications. Wang Y (186 articles), who reported two functional subsets of FOXP3+ Tregs in human thymus and periphery was the first in rank, followed by Sakaguchi S (164 articles), who reported the role of Tregs in immunity and identified FOXP3+ Tregs in the human immune system, and Zhang Y (153 articles), Liu Y (149 articles), and Li Y (140 articles), who focused on particular mechanisms and applications of Tregs. Each of the top 15 active authors contributed at least 90 articles to Treg research. Thus, they were called “productive authors.” However, the annual citation frequency of articles of these productive authors were not included in the top 15 with regard to annual citation frequency, except for those of Sakaguchi, suggesting that these productive authors should consider the quality and not only the quantity of their articles.

**Table 4:** The top 15 active authors, cited authors (CA), and cited references (CR) in regulatory T cell (Treg) research from 2000 to 2015.

Ranking	Freq	Author	Freq	CA	Freq (per year)	CR	Journal
1	186	Wang Y (HUST)	7,767	Sakaguchi S	227	Sakaguchi S (2008)	CELL V133 P775
2	164	Sakaguchi S	4,410	Fontenot JD	212	Hori S (2003)	SCIENCE V299 P1057
3	153	Zhang Y	3,671	Hori S	196	Fontenot JD (2003)	NAT IMMUNOL V4 P330
4	149	Liu Y	3,274	Shevach EM	171	Bettelli E (2006)	NATURE V441 P235
5	140	Li Y	2,289	Bettelli E	162	Vignali DAA (2008)	NAT REV IMMUNOL V8 P523
6	136	Zhang L	2,207	Thornton AM	134	Sakaguchi S (2004)	ANNU REV IMMUNOL V22 P531
7	134	Sparwasser T	2,159	Chen WJ	121	Curiel TJ (2004)	NAT MED V10 P942
8	121	Wang J (IBMS)	1,930	Curiel TJ	119	Coombes JL (2007)	J EXP MED V204 P1757
9	119	Wang L (Sichuan Univ)	1,717	Tang QZ	118	Sakaguchi S (2005)	NAT IMMUNOL V6 P345
10	112	Li J	1,684	Belkaid Y	118	Liu WH (2006)	J EXP MED V203 P1701
11	111	Bluestone JA	1,665	Jonuleit H	117	Chen WJ (2003)	J EXP MED V198 P1875
12	104	Blazar BR	1,598	Takahashi T	106	Fontenot JD (2005)	IMMUNITY V22 P329
13	91	Wang H (CAS)	1,507	Baecher-allanC	102	Khattri R (2003)	NAT IMMUNOL V4 P337
14	91	Lombardi G	1,447	Khattri R	87	Veldhoen M (2006)	IMMUNITY V24 P179
15	90	Wood KJ	1,402	Steinman RM	76	Shevach EM (2002)	NAT REV IMMUNOL V2 P389

Notes: HUST, Huazhong University of Science and Technology; IBMS, Institute of Basic Medical Sciences, China; CAS, Chinese Academy of Sciences.

**Fig 5:**
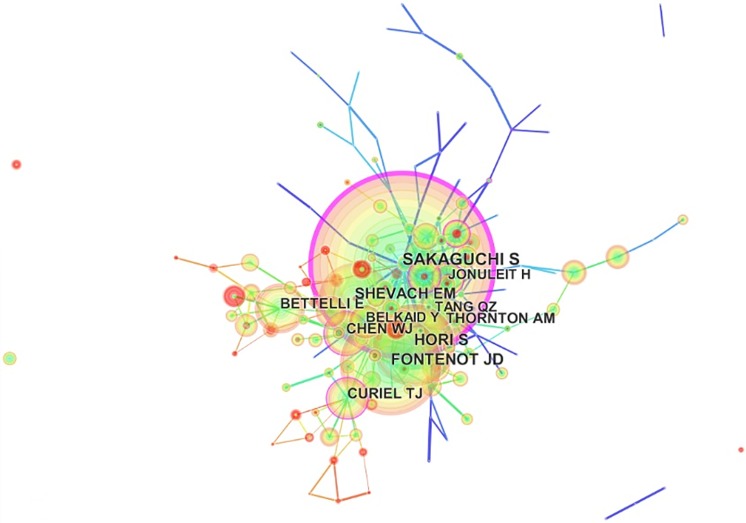
Co-citation map of authors who published articles related to regulatory T cell (Treg) research from 2000 to 2015.

Citation networks have been applied to information science analysis [29]. Here, we also analyzed author citations in Treg research using Citespace III and constructed related maps to estimate the scientific relevance of a publication. As shown in Fig 5 and Table 4, the largest nodes were associated with Sakaguchi (7,767 citations), Fontenot (4,410 citations), Hori (3,671 citations), and Shevach (3,274 citations), indicating their important role in Treg research. Additionally, Fig 5 revealed four large citation clusters; the first was typified by Sakaguchi, who initiated Treg research; the second by Hori and Fontenot, who focused on the development and plasticity of CD4+CD25+ Tregs by Foxp3; the third by Bettelli, who introduced the new area of Tregs and Th17; and the fourth by Curiel, who focused on the application of Tregs in ovarian carcinoma. These authors were from different disciplines and played important roles in Treg research.

### Cited reference cluster analysis

**Fig 6:**
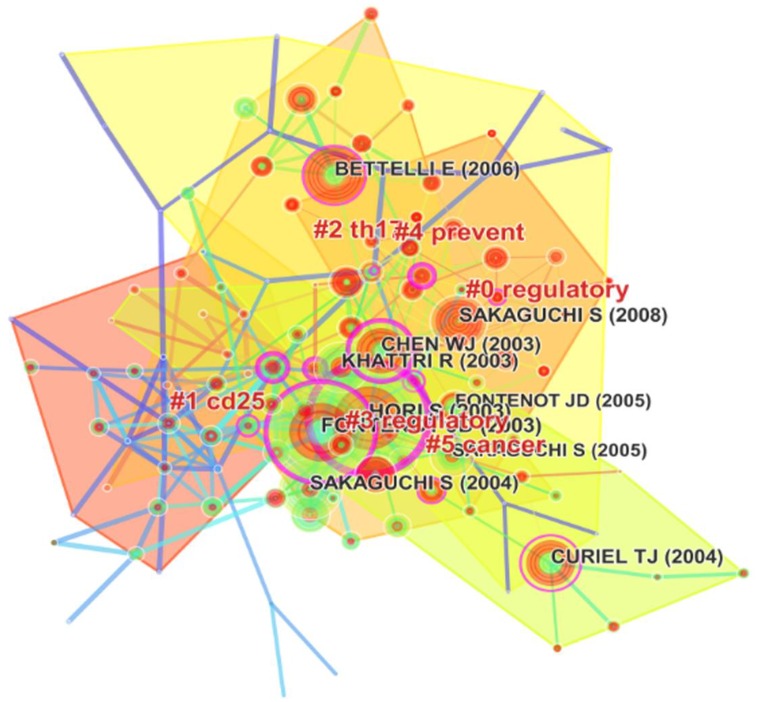
Reference co-citation map of articles related to regulatory T cell (Treg) research published from 2000 to 2015.

**Fig 7:**
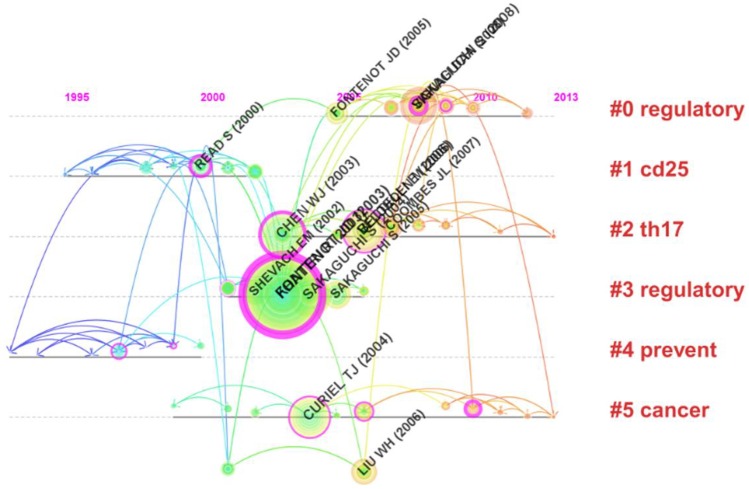
Reference co-citation time-view map of articles related to regulatory T cell (Treg) research published from 2000 to 2015.

Citation reference knowledge maps consist of references with higher centrality and citation counts [44]. The nodes represent different references, which are labeled with the years of publications and corresponding authors of cited references. The size of a cluster label is proportional to the size of the cluster [29]. Each cluster represents a distinct specialty or a thematic concentration [29] In the present study, we explored changes related to the key clusters of articles and constructed a cited reference map with 245 links, 136 nodes, and a silhouette value of > 0.5 (Figs 6 and 7). Among the 22 clusters, Cluster 3 (Treg) was the largest, whereas Cluster 1 (CD25) was the oldest. Cluster 3 included Hori (2003) [45], Sakaguchi (2004) [46], and Khattri (2003) [47] and formed the foundation of Treg knowledge. Cluster 1 included Read (2000) [48] and Shimizu (2002) [49], and reflected the onset stage of Treg research. Cluster 2 (th17) included Bettelli (2006) [50] and Coombes (2007) [51], who mainly investigated the balance of Treg and Th17. Cluster 5 included Curiel (2004) [52] and Zou (2006) [53], and reflected Treg-related therapy in cancer. As shown in Fig 7, most articles were published after 1995, with an especially high number in 2005. These results were in agreement with those shown in Fig 1.

We also analyzed the annual citation frequency of articles to compare the influence of a single paper. The results showed that an article that Sakaguchi S (2008) published in *Cell* had the highest number of citations (227 citations), suggesting that it is one of the most fundamental and important articles in Treg research, followed by articles published by Hori (2003; 212 citations) [47], Fontenot (2003; 196 citations), and Bettelli (2006; 171 citations) in *Nature Immunology*, *Nature*, and *Science* (Table 4). Additionally, *Nature Reviews Immunology*, *Journal of Experimental Medicine*, and *Immunity* also published some high-influence articles on Treg research. These articles are often considered to be fundamental in Treg research.

### Research area analysis

**Fig 8:**
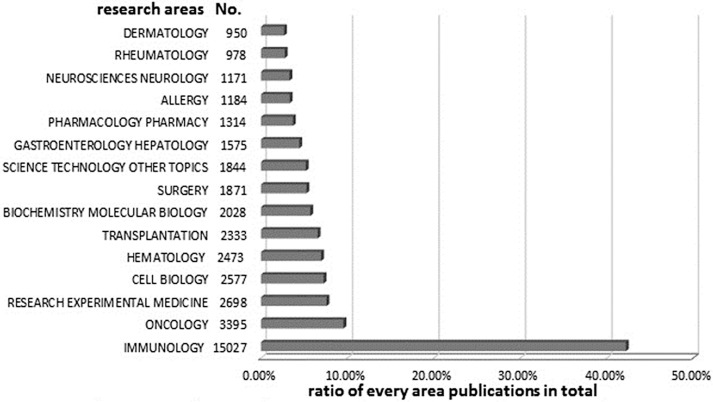
The 15 most frequently appearing research areas in regulatory T cell (Treg) studies from 2000 to 2015.

Extensive investigation of Tregs occurred in more than 100 special research areas. Fig 8 shows the 15 research areas that most frequently appeared in publications related to Treg research from 2000 to 2015. Here, the research areas (e.g., immunology, oncology, and cell biology) were defined as described in WoSCC [43]. Immunology accounted for the largest number of publications (42%), followed by oncology (10%), experimental medicine (5%), cell biology (7%), and hematology (7%).

### Keyword co-occurrence cluster analysis

**Fig 9:**
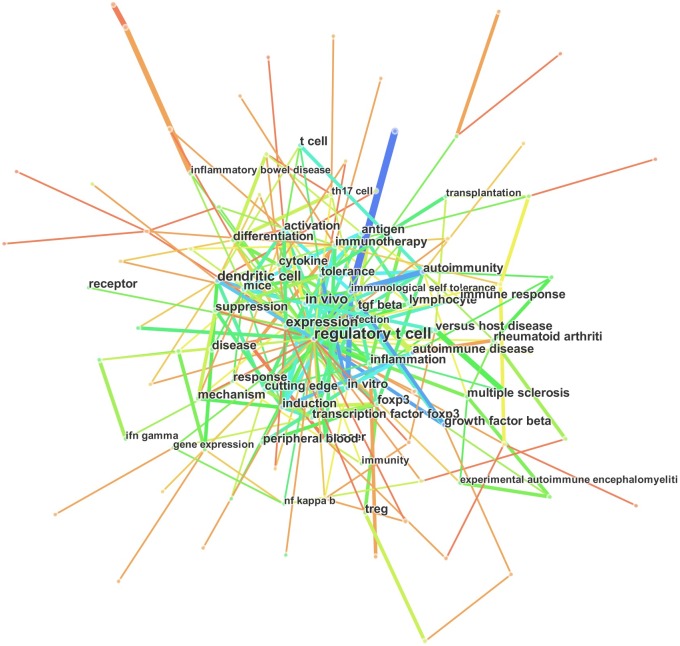
Map of keyword co-occurrence in articles related to regulatory T cell (Treg) research published from 2000 to 2015.

Keywords provide a reasonable description of research hotspots (focused attention by a number of scientific researchers to a set of related research problems and concepts), whereas burst words represent new research frontiers (emerging trends and abrupt changes that occur in a timely manner) [29]. In the present study, we used CiteSpace III to construct a knowledge map of keyword co-occurrence with 136 nodes and 245 links (Fig 9) and identified the top 20 keywords in Treg research articles from 2000 to 2015 (Table 5), according to frequency and citation counts.

**Table 5:** The 20 most frequently used keywords in articles related to regulatory T cell (Treg) research published from 2000 to 2015.

Ranking	Keyword	Frequency	Ranking	Keyword	Frequency
1	regulatory t cell	16,052	11	lymphocyte	1,718
2	dendritic cell	5,395	12	peripheral blood	1,709
3	gene expression	4,050	13	immunotherapy	1,683
4	in vivo	3,839	14	cytokine	1,662
5	tolerance	3,024	15	autoimmune disease	1,542
6	immune response	2,753	16	rheumatoid arthritis	1,311
7	inflammation	2,482	17	cancer	1,298
8	tgf beta	2,424	18	multiple sclerosis	1,291
9	autoimmunity	2,326	19	transplantation	979
10	foxp3	2,220	20	Th17 cell	851

#### Research hotspots

The top keywords were ‘regulatory T cell,’ ‘dendritic cells,’ ‘expression,’ ‘*in vivo*,’ ‘tolerance,’ ‘immune response,’ and ‘inflammation.’ Therefore, the top four hotspots of Treg research were:

1. Foxp3: Foxp3 is the most sensitive marker of Tregs and controls their function according to the expression of transcription factors; thereby defining their biology [54]. Foxp3 regulates the ‘desired’ gene expression profile of Tregs, and the loss of Foxp3 triggers ‘non-signature’ effector-like gene expression and ablates suppressive function [55], partly through control by demethylation.
2. Gene expression: In addition to epigenetic and transcriptional regulation, Treg function and T-cell immune response are also controlled by microRNA (miRNA)-dependent mechanisms. miRNAs are expressed from endogenous genes and their levels increase with the processing of primary transcripts mediated by Dicer and Drosha [54]. The expression of target gene products is suppressed by miRNAs through the RNA-induced silencing complex, which suppresses the degradation or translation of transcripts [56].
3. Cytokine/chemokine/induction/Th17: Tregs, as a subset of T cells, change their phenotype mainly via external or internal cytokines (e.g., IL-2, TGF-β, IL-6, TNF-α, IL-10, IL-1β, IL-17, and IL-35) and chemokines (e.g., CCL3), which bind to membrane receptors (TLR, GITR, and TCR) of Tregs and activate STAT3, WNT, and PI3K/AKT signaling [3, 54, 57–62]. Lack of balance in these factors results in Treg conversion into other T cells such as iTregs and Th17 cells [54].
4. Autoimmunity/inflammation disease: Tregs play an essential role in the prevention of autoimmune diseases and the maintenance of peripheral tolerance [1, 63] in cancer therapy [15, 64], transplantation tolerance [9, 19, 65, 66], inflammatory bowel disease [67], allergy/asthma [68, 69], liver disease [70], blood diseases [71], and dermatological diseases [72].

#### Research frontiers

**Fig 10:**
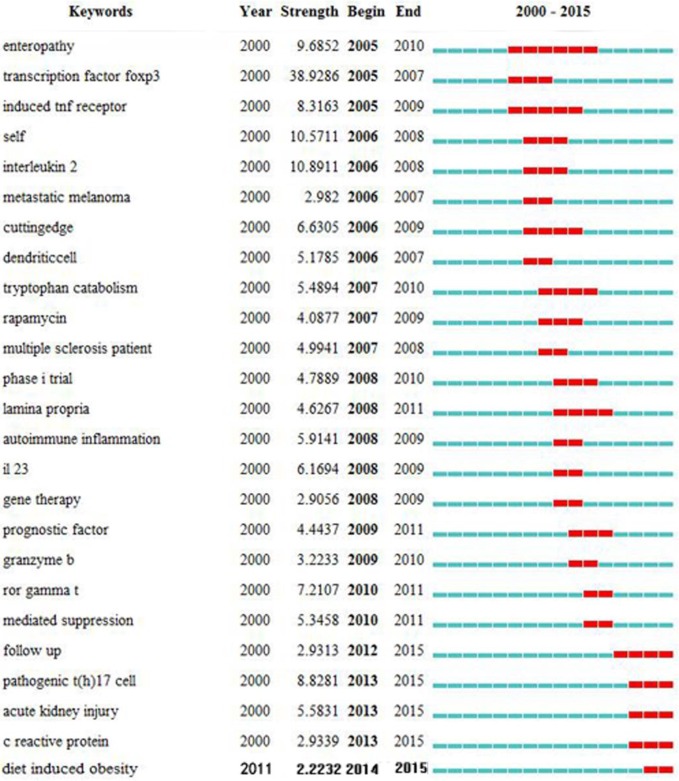
Top 25 keywords with the strongest citation bursts in articles related to regulatory T cell (Treg) research published from 2000 to 2015.

We used CiteSpace III to detect burst keywords, which are considered indicators of research frontiers over time. We depicted the time interval as a blue line and the time period that represents a burst keyword category as a red line, indicating the beginning and the end of the time interval of each burst [26, 73]. As shown in Fig 10, the top four frontiers of Treg research were:

1. Autoimmune inflammation: Since their discovery, the functions of Tregs in immunity and inflammation have been studied extensively [74–76].
2. RORγt and Th17: RORγt is a transcriptional promoter that plays a pivotal role in Th17 cell formation [20]. Th17 and Treg cells have opposing functions in immunoregulation, and changes in their ratio have provided novel therapeutic strategies [75].
3. Granzyme B: Treg suppression is mediated by the extracellular and non-apoptotic activity of granzyme B [77]. Reduced numbers of Tregs in some patients have functional consequences as a result of exaggerated T cell responses [78].
4. Gene therapy: Tregs are subsets of T lymphocytes specialized in the modulation of antigen-specific immune responses *in vivo*. Hence, Tregs represent an ideal therapeutic tool to control detrimental immune reactions [79]. Treg gene therapy has gradually become an important therapeutic strategy for some immunity-related conditions, including cancer [80, 81], organ transplantation [82], and acute kidney injury, [83], and further research continues for other diseases [79].

## Conclusions

Here, we investigated the global scientific outputs of Treg research from 2000 to 2015, analyzing data on publication outputs, countries, journals, authors, institutions, and research areas obtained from the WoSCC. Our objective was to identify Treg research trends and hotspots. In summary, we observed that the number of publications increased with time since 2000. *The Journal of Immunology* published the highest number of articles on Treg research, followed by *Plos One*, *Blood*, *American Journal of Transplantation*, *European Journal of Immunology*, and *Immunology*. The USA and China were the top countries for Treg research, followed by Germany, UK, and Japan. Collaborations between institutions were seldom observed and need to be enhanced in the future. The highest-impact scholars were Sakaguchi, Fontenot, Hori, Shevach, and Wang Y (HUST). Sakaguchi (2004, 2008), Hori (2003), Fontenot (2003, 2005), and Bettelli (2006) had the most cited publications. Immunology was the most prevalent research area, followed by oncology, experimental medicine, cell biology, and hematology. The results showed that the hotspots of Treg research were dendritic cells, gene expression, immune tolerance, foxp3, inflammation, and immunotherapy, whereas the frontiers of research were autoimmune inflammation, gene therapy, granzyme b, and RORγt.

In summary, this bibliometric analysis revealed that Treg-related studies are still hotspots, and that Treg-related clinical therapy is a research frontier; however, further studies and more collaborations are needed in this field of study worldwide. Overall, our findings might provide valuable information for the editors of immunology journals to identify new perspectives and shape future research directions.
