# Correction: Global Regulatory T-Cell Research from 2000 to 2015: A Bibliometric Analysis

**DOI:** 10.1371/journal.pone.0170850

**Published:** 2017-01-23

**Authors:** Yin Zongyi, Chen Dongying, Li Baifeng

There is an error in the sixth sentence of the Abstract. The correct sentence is: Sakaguchi, Rudensky, Hori, Shevach, and Bettelli were the top authors in Treg research.

There is an error in the third sentence in the second paragraph of the Author analysis section of the Results and Discussion. The correct sentence is: As shown in Fig 5 and Table 4, the largest nodes were associated with Sakaguchi (8,390 citations), Rudensky (4,748 citations), Hori (3,971 citations), and Shevach (3,554 citations), indicating their important role in Treg research.

There is an error in the image for Fig 5. Please see the correct Fig 5 here.

**Fig 5:**
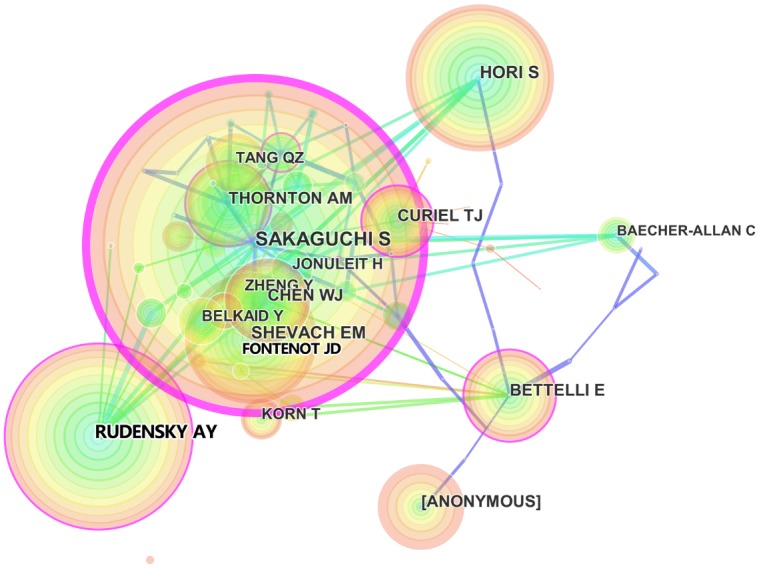
Co-citation map of authors who published articles related to regulatory T cell (Treg) research from 2000 to 2015.

The values in the Freq column of Table 4 are incorrect. Please see the correct Table 4 here.

**Table 4:** The 15 most active Authors, Cited-Authors and Cited Reference.

Ranking	Freq	Author	Freq	CA	Freq (/year)	CR	Journal
1	186	Wang Y (HUST)	8390	Sakaguchi S	227	Sakaguchi S(2008)	CELL V133 P775
2	164	Sakaguchi S	4748	Rudensky AY	212	Hori S(2003)	SCIENCE V299 P1057
3	153	Zhang Y	3971	Hori S	196	Fontenot JD(2003)	NAT IMMUNOL V4 P330
4	149	Liu Y	3554	Shevach EM	171	Bettelli E(2006)	NATURE V441 P235
5	140	Li Y	2417	Bettelli E	162	Vignali DAA(2008)	NAT REV IMMUNOL V8 P523
6	136	Zhang L	2373	Thornton AM	134	Sakaguchi S(2004)	ANNU REV IMMUNOL V22 P531
7	134	Sparwasser T	2317	Fontenot JD (UW)	121	Curiel TJ(2004)	NAT MED V10 P942
8	121	Wang J (IBMS)	2217	Chen WJ	119	Coombes JL(2007)	J EXP MED V204 P1757
9	119	Wang L (Sichuan Univ)	1965	Curiel TJ	118	Sakaguchi S(2005)	NAT IMMUNOL V6 P345
10	112	Li J	1606	Tang QZ	118	Liu WH(2006)	J EXP MED V203 P1701
11	111	Bluestone JA	1398	Belkaid Y	117	Chen WJ(2003)	J EXP MED V198 P1875
12	104	Blazar BR	1296	Zheng Y	106	Fontenot JD(2005)	IMMUNITY V22 P329
13	91	Wang H (CAS)	1193	Korn T	102	Khattri R(2003)	NAT IMMUNOL V4 P337
14	91	Lombardi G	1114	Jonuleit H	87	Veldhoen M(2006)	IMMUNITY V24 P179
15	90	Wood KJ	1087	Baecher-allan C	76	Shevach EM(2002)	NAT REV IMMUNOL V2 P389

Note: CA: Cited-Authors; CR: Cited Reference; HUST: Huazhong University of Science and Technology; IBMS: Institute of Basic Medical Sciences, China; CAS: Chinese Academy of Sciences; UW: University of Washington, Seattle.

There is an error in the fourth sentence in the second paragraph of the Author analysis section of the Results and Discussion. The correct sentence is: Additionally, Fig 5 revealed four large citation clusters; the first was typified by Sakaguchi, who initiated Treg research; the second by Hori and Rudensky, who focused on the development and plasticity of CD4+CD25+ Tregs by Foxp3; the third by Bettelli, who introduced the new area of Tregs and Th17; and the fourth by Curiel, who focused on the application of Tregs in ovarian carcinoma.

There is an error in the seventh sentence of the Conclusion. The correct sentence is: The highest-impact scholars were Sakaguchi, Rudensky, Hori, Shevach, and Bettelli.
